# Sodium Iodate-Induced Degeneration Results in Local Complement Changes and Inflammatory Processes in Murine Retina

**DOI:** 10.3390/ijms22179218

**Published:** 2021-08-26

**Authors:** Anne Enzbrenner, Rahel Zulliger, Josef Biber, Ana Maria Quintela Pousa, Nicole Schäfer, Corinne Stucki, Nicolas Giroud, Marco Berrera, Elod Kortvely, Roland Schmucki, Laura Badi, Antje Grosche, Diana Pauly, Volker Enzmann

**Affiliations:** 1 Department of Ophthalmology, University Hospital Regensburg, 93053 Regensburg, Germany; a.enzbrenner@googlemail.com (A.E.); Nicole.Schaefer@klinik.uni-regensburg.de (N.S.); 2 Roche Pharma Research & Early Development, Roche Innovation Center Basel, F. Hoffmann-La Roche Ltd., 4070 Basel, Switzerland; rahel.zulliger@roche.com (R.Z.); corinne.stucki@roche.com (C.S.); nicolas.giroud@roche.com (N.G.); marco.berrera@roche.com (M.B.); elod.koertvely@roche.com (E.K.); roland.schmucki@roche.com (R.S.); laura.badi@roche.com (L.B.); 3 Department of Physiological Genomics, Biomedical Center, Ludwig-Maximilians-University Munich, 82152 Planegg-Martinsried, Germany; Josef.Biber@bmc.med.lmu.de (J.B.); Antje.Grosche@bmc.med.lmu.de (A.G.); 4 Department of Ophthalmology, University Hospital of Bern, 3010 Bern, Switzerland; quintelapousa@gmail.com (A.M.Q.P.); volker.enzmann@insel.ch (V.E.); 5 Department of Biomedical Research, University of Bern, 3010 Bern, Switzerland; 6 Experimental Ophthalmology, University Marburg, 35043 Marburg, Germany

**Keywords:** retinal degeneration, geographic atrophy, sodium iodate, local complement, inflammation, innate immunity, mouse

## Abstract

Age-related macular degeneration (AMD), one of the leading causes of blindness worldwide, causes personal suffering and high socioeconomic costs. While there has been progress in the treatments for the neovascular form of AMD, no therapy is yet available for the more common dry form, also known as geographic atrophy. We analysed the retinal tissue in a mouse model of retinal degeneration caused by sodium iodate (NaIO_3_)-induced retinal pigment epithelium (RPE) atrophy to understand the underlying pathology. RNA sequencing (RNA-seq), qRT-PCR, Western blot, immunohistochemistry of the retinas and multiplex ELISA of the mouse serum were applied to find the pathways involved in the degeneration. NaIO_3_ caused patchy RPE loss and thinning of the photoreceptor layer. This was accompanied by the increased retinal expression of complement components *c1s, c3*, *c4*, *cfb* and *cfh*. C1s, C3, CFH and CFB were complement proteins, with enhanced deposition at day 3. C4 was upregulated in retinal degeneration at day 10. Consistently, the transcript levels of proinflammatory *ccl-2*, *-3*, *-5*, *il-1β*, *il-33* and *tgf-β* were increased in the retinas of NaIO_3_ mice, but *vegf-a* mRNA was reduced. Macrophages, microglia and gliotic Müller cells could be a cellular source for local retinal inflammatory changes in the NaIO_3_ retina. Systemic complement and cytokines/chemokines remained unaltered in this model of NaIO_3_-dependent retinal degeneration. In conclusion, systemically administered NaIO_3_ promotes degenerative and inflammatory processes in the retina, which can mimic the hallmarks of geographic atrophy.

## 1. Introduction

Dysfunctional retinal pigment epithelium (RPE) and degenerated neurosensory retina play a central role in the pathobiology of age-related macular degeneration (AMD), the leading cause of blindness among the elderly in Western societies [1]. The oxidative events and inflammation drive the pathological changes in the retina and RPE during degeneration [2,3]. This is seen in animal studies, which were performed to elucidate the molecular interplay of oxidative stress and tissue-specific complement activation in the eyes [4]. Furthermore, after the discovery of genetic risk variants in several complement pathway genes, the innate immunity was emphasised as an important mechanism in AMD [5]. Thereby, diverse components of the immune system, including resident microglia and recruited macrophages, inflammatory activators and pathways, especially from the complement cascade, as well as an activated inflammasome, are involved [6]. The sodium iodate (NaIO_3_) model mimics the aspects of geographic atrophy [7]. Upon systemic administration, NaIO_3_ specifically targets the RPE, resulting in its patchy loss via necrosis/necroptosis [8,9] and subsequent apoptosis of adjacent photoreceptors [7,10]. Oxidative stress and multiple caspase-dependent and -independent cell death pathways have been identified in retinal degeneration induced by NaIO_3_ [11]. Furthermore, the increased expression of tissue modulators, including stromal cell-derived factor-1 (SDF-1), hepatocyte growth factor (HGF) and leukaemia inhibitory factor (LIF), have been found in NaIO_3_-affected retinas [12]. A cellular response of the immune system, including the activation of macrophages, is involved as well [13]. Furthermore, the complement system as a pathway of the innate immune system has been observed to be upregulated after NaIO_3_ treatment in vitro and in vivo [14,15].

In this study, a patchy RPE loss causing photoreceptor apoptosis was associated with the accumulation of classical (C1s and C4) and alternative (CFH and CFB) complement components in the retina, converging in the modulation of the complement pathway, as indicated by the accumulation of C3 in the retinal tissue. In addition, a local rise in chemokines was observed. Systemic inflammatory changes could not be detected. In sum, this suggested a retinal and not a blood-derived origin of the local rise in inflammatory mediators, even though NaIO_3_ was administered systemically. Microglia, monocytes and gliotic Müller cells could be the main source of inflammatory proteins in the retina.

## 2. Results

### 2.1. NaIO_3_-Induced Retinal Degeneration

**Figure 1:**
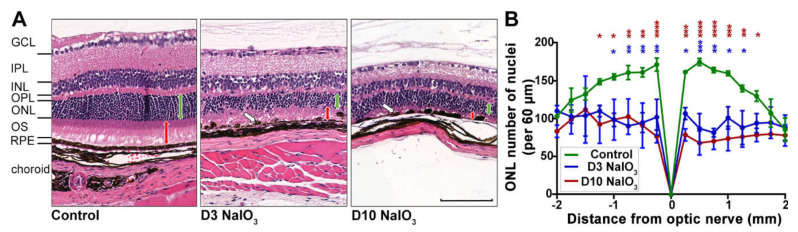
Time course of NaIO_3_-induced retinal degeneration. (**A**) H&E staining of murine retinal cross-sections at three (D3) and ten (D10) days following the NaIO_3_ treatment showed a disruption of the RPE layer, clustering of the RPE cells (white arrows) and photoreceptor degeneration. The photoreceptor segments (PS) were reduced at three days and nearly completely missing ten days after the treatment (red arrows), but also, the thickness of the outer nuclear layer (ONL) decreased substantially (green arrows). NFL, nerve fibre layer; GCL, ganglion cell layer; IPL, inner plexiform layer; INL, inner nuclear layer; OPL, outer plexiform layer. Scale bar: 50 µm. (**B**) The number of photoreceptor nuclei per 60-µm retinal section was counted to quantify the progress of degeneration. A two-way ANOVA with Bonferroni post-tests showed reduced photoreceptor numbers in treated versus untreated mice (* *p* < 0.05, ** *p* < 0.01, *** *p* < 0.001 and **** *p* < 0.0001).

To investigate the immunological consequences of damaged RPE in a mouse model of retinal degeneration, systemic NaIO_3_ administration was used to disrupt the integrity of the RPE cell layer (Figure 1). Due to a high variability in previous studies using NaIO_3_ and the varying oxidising potency of this agent, a confirmation of retinal degeneration for each NaIO_3_ study cycle was needed. In the present study, the intravenous application of NaIO_3_ resulted in the formation of RPE cell agglomerations (blebs) at day 3 and RPE disruption at day 10 (Figure 1A).

As a secondary effect, degeneration of the photoreceptor cells was observed (Figure 1), as they depend on an intact RPE to maintain their physiological metabolism. On day 3 following the treatment, the photoreceptor segments decreased in length, and at day 10, they were hardly detectable (Figure 1A). The number of photoreceptor nuclei significantly decreased in the NaIO_3_-treated mice at day 3 and continued at day 10 in the central retina, while their number in the untreated control mice did not change (Figure 1B).

However, when evaluated longitudinally at the full length, the pattern of degeneration within a single retina was observed to be nonuniform (Supplementary Figure S1A): the zones of normal retinal morphology alternated with the areas of RPE and photoreceptor degeneration, a feature of NaIO_3_ treatment commonly described as “patchy RPE loss” [16,17,18]. Further, the blood–retinal barrier was at least partly intact, as antibodies penetrating from the systemic bloodstream into the retinal tissue were not observed (Supplementary Figure S1B).

### 2.2. NaIO_3_ Treatment Increased Retinal Complement mRNA

**Figure 2:**
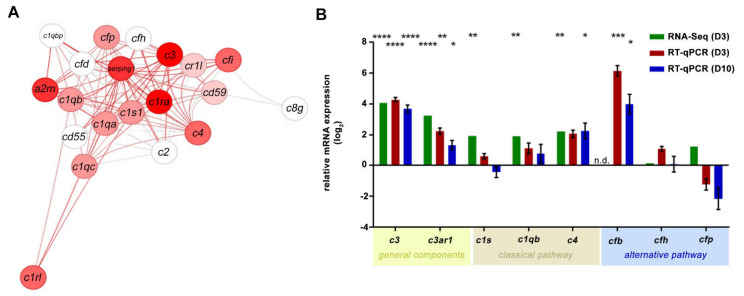
Increase in the mRNA expression of the complement components in the retina following NaIO_3_ treatment. (**A**) The transcript quantity of the components of the complement pathway (GO: 0006956) was changed in the retinas after the NaIO_3_ treatment at day 3. Nodes are the genes with their connections as defined in STRING database version 10, and the colour is given by the fold change (log2FC = 4) of the regulation (red = up, white = no regulation). (**B**) The fold change (log_2_) mRNA expression levels of the general (*c3* and *c3ar1*), classical (*c1s* and *c4*) and alternative (*cfb*) pathway components were analysed in independent experiments using either RNA-seq or RT-qPCR. The retinal tissue was harvested at day 3 (RNA-seq analysis, n = 5) or day 3 and day 10 (RT-qPCR, n = 6) after NaIO_3_ application. RNA-seq gene expression was normalised to the overall gene set. The RT-qPCR expression was normalised to the *idh3b* reference mRNA. Fold change in the expression (compared to the untreated control, line at y = 0) was calculated on the logarithm to base 2. The gene expressions of treated versus untreated retinas were evaluated using ordinary one-way ANOVA and Tukey’s multiple comparisons test (* 0.01 < *p* < 0.05, ** 0.001 < *p* < 0.01, *** 0.0001 < *p* < 0.001 and **** *p* < 0.0001).

Systemically applied NaIO_3_ caused degeneration of the RPE and subsequent retinal degeneration. We then aimed to assess the associated inflammatory effects of the NaIO_3_ treatment in the murine neurosensory retina. The RNA-seq analysis in the retinas revealed an upregulation of multiple complement component mRNAs in NaIO_3_ retinas compared to those of the controls (Figure 2A and Supplementary Figure S2).

The influence of NaIO_3_ on the expression of complement genes was confirmed in RT-qPCRs from isolated retinas, showing a consistent complement mRNA increase, including that of *c3*, *c3ar1*, *c1s* and *c4* in NaIO_3_ mice compared to the controls on day 3 (Figure 2B). The *c1qb* and *cfp* transcripts were only significantly upregulated in the RNA-seq evaluation. In contrast, the *cfb* levels were significantly increased in the RT-qPCR experiments but were not detected in the RNA-seq analysis. To investigate the more long-term effects of NaIO_3_; in addition to day 3, day 10 post-treatment was also evaluated by RT-qPCR (Figure 2B). Most components upregulated three days post-NaIO_3_ were still expressed at significantly higher levels than in the control retinas, except for *c1s* mRNA, which returned to the control levels on day 10. 

### 2.3. NaIO_3_ Treatment Promoted Retinal Complement Deposition

**Figure 3:**
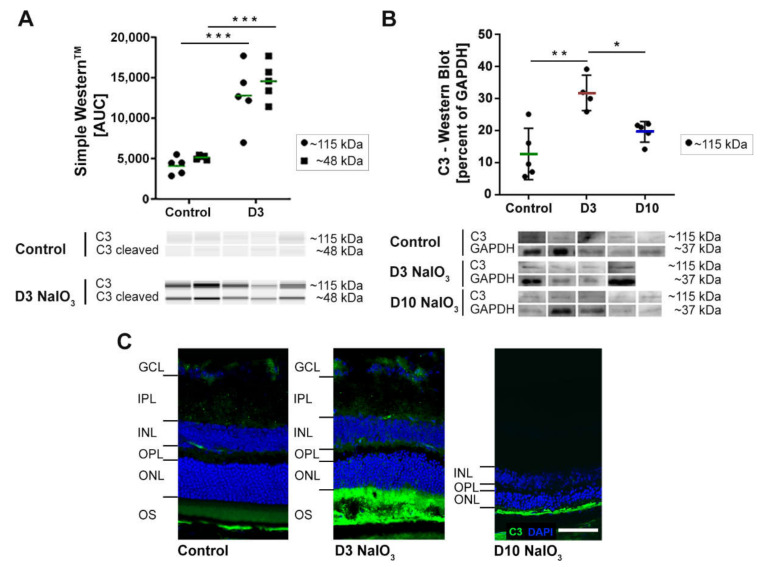
The C3 protein signals in murine neurosensory retina were increased after the NaIO_3_ treatment. (**A**) The protein levels of the complement component C3 were significantly increased at day 3 after the NaIO_3_ treatment in all the samples compared to the untreated control using Simple Western^TM^ technology. Full blots are shown in Supplementary Figure S3A. (**B**) The Western blot signals of C3 in the retinal protein extracts of NaIO_3_-treated mice under waning conditions were increased three days after treatment. The C3 protein levels were comparable to the untreated controls at day 10. GAPDH was used as a housekeeping protein. The intensity of the C3 signal in 3A and 3B was measured using ImageJ software. * 0.01 < *p* < 0.05, ** 0.001 < *p* < 0.01 and *** 0.0001 < *p* < 0.001 (one-way ANOVA, Tukey’s multiple comparisons test). The full blots are shown in Supplementary Figure S3B. (**C**) The immunohistochemical stainings of C3 and DAPI three days and ten days after the NaIO_3_ treatment showed C3 accumulation in the POS at day 3, whereas the signal decreased after ten days. At this point in time, the ONL and PS thickness were reduced (see, also, Figure 1). Scale bar: 50 µm.

C3, the complement component that is central to all three complement pathways, was analysed at the protein level to confirm the RNA expression results (Figure 3). Comparative Western and Simple Western™ blots were performed on the retinal protein extracts in order to accommodate the different sample availability. Thereby, C3 was detected at ~115 kDa in all the neurosensory retina samples of both untreated and NaIO_3_-treated mice. We confirmed increased C3 protein levels in NaIO_3_-treated mouse retinas at day 3 (Figure 3A,B). However, and in contrast to the RNA data (Figure 2), the C3 protein signals returned to the baseline levels at day 10. The immunohistochemical staining (IHC) of C3 revealed an accumulation of proteins at the photoreceptor outer segment (POS) layer of NaIO_3_-treated mouse retinas (Figure 3C). This distinct C3 accumulation was only present on day 3. Ten days post-treatment, a faint staining remained detectable in conjunction with almost completely degenerated POS (see Figure 1). 

**Figure 4:**
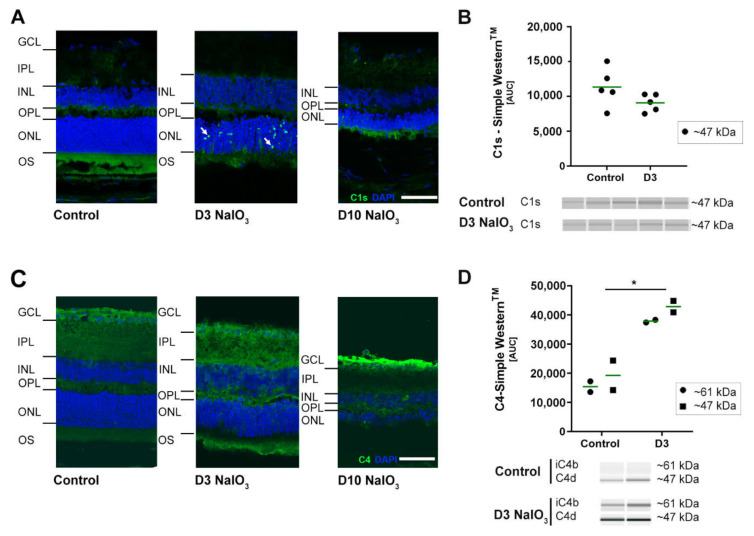
Classical pathway proteins C1s and C4 accumulated in NaIO_3_ damaged the retinas. (**A**) C1s immunoreactivity (green) was localised (white arrows) to the ONL at three days after the NaIO_3_ treatment. At ten days, no specific C1s staining remained visible. DAPI staining (blue) delineated cell nuclei. (**B**) C1s protein levels were not changed in the Simple Western^TM^ three days after the NaIO_3_ treatment. (**C**) C4 (green) was observed in the nerve fibre layer (NFL) ten days following the NaIO_3_ treatment. In contrast, no distinct C4 staining was visible at day 3. Scale bar: 50 µm. (**D**) C4 cleavage products C4d (47 kDa) and iC4b (61 kDa) were increased at day 3 after the NaIO_3_ treatment in all samples compared to the untreated control using Simple Western^TM^ technology. (**B**,**D**) Intensity of the signals was measured using ImageJ software. * 0.01 < *p* < 0.05 (one-way ANOVA and Tukey’s multiple comparisons test). The full blots are shown in Supplementary Figure S3C,D.

To determine whether NaIO_3_ also enhances the systemic activation of the complement system, levels of the anaphylatoxin C3a were evaluated in mouse serum by ELISA (Supplementary Figure S4), but no changes of the systemic levels of C3a were detectable ten days following intravenous NaIO_3_ injection compared to untreated mice (serum of day 3 was not available). Additionally, to the increased expression of complement component C3 post-NaIO_3_ treatment; also, the mRNA of the classical/lectin pathway components *c1s* and *c4* were significantly increased (Figure 2). This corresponded to the respective protein levels in IHC stainings in the murine retina (Figure 4A,C).

Three days after the treatment, the classical pathway initiator protein C1s were selectively upregulated in the ONL, apparently close to the photoreceptor nuclei (Figure 4A). Ten days post-NaIO_3_, C1s staining disappeared, but C4 accumulated in the innermost part of the retina, the ganglion cell layer and nerve fibre layer, respectively (Figure 4C). Simple Western™ studies confirmed C4d and iC4b accumulation in the retina three days after NaIO_3_ (Figure 4B,D, respectively) but did not disclose the changes in the C1s levels shown with IHC. 

**Figure 5:**
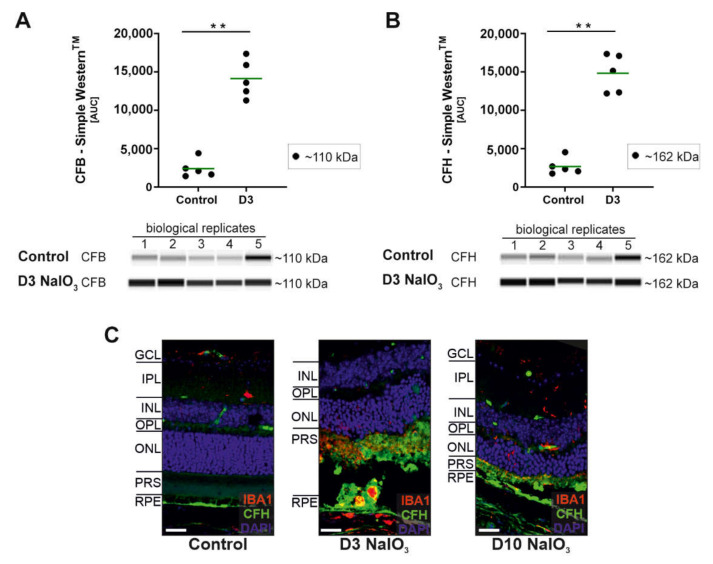
Alternative pathway component CFB and CFH protein levels were increased after the NaIO_3_ treatment. (**A**) The CFB (~110 kDa) protein levels were increased at day 3 after the NaIO_3_ treatment in all the samples compared to an untreated control using Simple Western^TM^ technology for detection. (**B**) Complement inhibitor CFH (~162 kDa) accumulated in the neurosensory retina of NaIO_3_-treated mice compared to the control. (**C**) Retinal degeneration using NaIO_3_ resulted in CFH deposition in the photoreceptor outer segment layer shown in immunohistochemical stainings. An overlapping with microglia marker IBA-1 was observed. (**A**,**B**) GAPDH was used as a housekeeping protein. The intensity of the protein signal was measured using ImageJ software. ** 0.001 < *p* < 0.01 (unpaired, nonparametric Mann–Whitney *t*-test). The full blots are shown in Supplementary Figure S3E,F.

Finally, changes in the mRNA levels of *cfb* (significant increase) but not for *cfh* (not a significant increase) were observed in NaIO_3_-treated mice (Figure 2). Both are components of the alternative pathway. The protein analysis confirmed these findings for CFB but not for CFH (Figure 5), as both proteins were significantly increased in the retinas three days after the systemic NaIO_3_ application.

**Figure 6:**
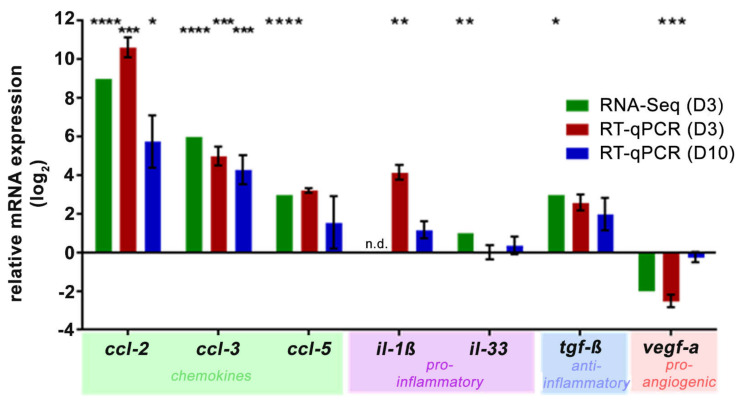
NaIO_3_ increased the retinal transcription of the chemokines and cytokines. RNA-seq (day 3, green) and RT-qPCR analysis (day 3, red; day 10, blue) of the *ccl-2, 3, 5, il-1β and tgf-β* expression showed a significant upregulation after the NaIO_3_ treatment. *Il-33* was not significantly upregulated, whereas *vegf-a* was decreased on the mRNA level at day 3 following the treatment. The expression was normalised to the overall gene set (RNA-seq) or *idh3b* reference mRNA (RT-qPCR), and the fold change in the expression (compared to the untreated control, line at y = 0) was calculated on the logarithm to base 2. * 0.01 < *p* < 0.05, ** 0.001 < *p* < 0.01, *** 0.0001 < *p* < 0.001 and **** *p* < 0.0001 (ordinary one-way ANOVA and Tukey’s multiple comparisons test).

In addition to the complement component analysis, the systemic NaIO_3_ treatment had a local effect on the retinal chemokine/cytokine status. Chemokines *ccl-2* and *ccl-3* were highly upregulated three and ten days after NaIO_3_ in the RNA-seq and RT-qPCR analyses (Figure 6), and a similar trend was observed for *ccl-5*. The expression of proinflammatory cytokine *il-1β* increased compared to the control levels in one of the two transcription determining techniques, and for *il-33*, minimal changes were also determined. Consistently, the mRNA levels (as determined by RNA-Seq and RT-qPCR) of the multifunctional cytokine *tgf-β* showed an upward trend in response to NaIO_3_, whereas *vegf-a*, a proangiogenic factor, was reduced three days after the NaIO_3_ treatment and returned to the control levels on day 10. 

To further evaluate to what extent intravenously applied NaIO_3_ caused systemic inflammatory dysregulation, the serum cytokine protein levels were determined by multiplex ELISA (Supplementary Figure S5). For the tested chemokines (CCL-2, CCL-3 and CCL-5) and cytokines/growth factors (IL-1β and VEGF-A), no significant alteration of the serum concentrations ten days after NaIO_3_ treatment were observed. 

**Figure 7:**
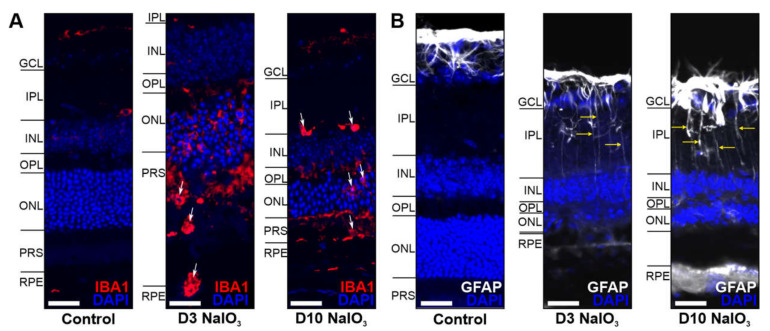
Glia cells were activated in the NaIO_3_-damaged retina. (**A**) Microglia/monocytes (arrows) migrated into the photoreceptor outer segment layer at day 3 following the NaIO_3_ treatment and were localised in the IPL at day 10. (**B**) GFAP (white), a marker for activated Müller cells, was enhanced in the GCL after NaIO_3_ treatment during the investigated period. Scale bar: 20 µm.

Increased inflammatory signalling in the retina is often associated with microglia activation, monocyte invasion and Müller cell gliosis. Indeed, we found subretinal microglia/monocytes associated with the PR outer segments three days after treatment (Figure 7A). The outer segments seem to become opsonised by C3b (compared to Figure 4) and phagocytised. After ten days, the outer segments were almost completely degraded, and no IBA1-positive cells were present in this region anymore. Müller cell gliosis at day 10 was a consequence in the damaged retinas (Figure 7B). 

In summary, systemic NaIO_3_ application caused damage of the RPE and, subsequently, the neurosensory retina involving proinflammatory signalling via the local complement components and chemokines. Thereby, early complement proteins such as C3, C1s, CFH and CFB showed an increased deposition at day 3, whereas the late complement component C4 was involved in retinal degeneration at day 10.

## 3. Discussion

### 3.1. Validation of the NaIO_3_-Induced Retinal Degeneration Model 

Systemically applied NaIO_3_ has been frequently used to investigate murine retinal degeneration. While the cause of morphological changes has been quite well-described over the past few decades [7], research defining the inflammatory consequences of NaIO_3_ treatment has only come into focus recently [12,15].

The impact of different doses of NaIO_3_ in mice has been extensively described in the literature [10,19]. The main conclusion was that a low dose only has a temporary effect on the function of the retina and does not lead to extensive changes in the morphology. An intermediate dose has been described to cause a “patchy” RPE cell loss with a subsequent degeneration of the photoreceptor cells, while a large dose inevitably leads to complete destruction of the retinal structure and function in a short amount of time. Variations between published studies are due to different application methods (intraperitoneal or intravenously) but also due to the unstable character of the chemical, NaIO_3_, itself. Therefore, it is important that each study proves the potency of the NaIO_3_ batch before any treatment studies are performed. We could confirm the patchy RPE cell loss, as well as the degeneration of the overlying photoreceptors in our model. A validated intermediate dose was then used for the analysis of the complement pathway and other inflammatory components.

### 3.2. The Complement System in the Eye and Its Involvement in NaIO_3_-Induced Retinal Degeneration

As the complement system is an integral part of the innate immune system, it is also involved in inflammatory processes causing retinal degeneration in diseases like AMD, glaucoma and diabetic retinopathy. In the eye, as a partially immune-privileged compartment of the human body with a tight blood–retina barrier, the ocular tissue is required to produce a large number of innate immune system proteins itself, while other organs can rely on the supply from the liver [20]. This is especially true for the many components of the complement system, which, in fact, is a crucial pillar for innate immunity [21]. In line with this, our analyses of the retinal tissue in the acute phase of the NaIO_3_ treatment induced retinal degeneration and showed a significant upregulation of complement components. We conclusively demonstrated that the central complement protein C3 was significantly upregulated at the transcript and protein levels upon the NaIO_3_ treatment—a finding that served as a quality control for our model and was complementary to previous studies [12,13,15,22]. In addition, while C3 is the central connecting factor of the complement cascade, it does not lead to inflammatory processes by itself, and its interaction with additional complement proteins is always needed. We demonstrated recently that mainly complement components of the classical and the alternative pathways are expressed in healthy mouse retinal tissue [21], while the components of the lectin pathway (e.g., MASP1) were only expressed at very low levels or were not detectable at all. We could confirm these findings with our presented RNA-seq analysis. Interestingly, CFB was only detected by qPCR and not in RNA-seq, but as others described its presence in the mouse retina, the lack of detection in our analysis might be due to different technical preferences [23]. However, there is also evidence that the liver serves as the main source of ocular CFB, and very little if any expression is found in healthy eyes [24].

In the classical pathway, the initial components leading to an activation of the downstream cascade are C1q, C1s and C1r. In the RNA-seq analysis of the retinal tissue, these C1 complex components were, in fact, upregulated after the treatment with NaIO_3_. For *c1s*, these results were also confirmed by qPCR, and via immunostaining, an accumulation at the ONL was demonstrated. Another crucial component of the classical pathway downstream of C1 is C4, whose expression in the retina was also significantly increased after NaIO_3_ application. Katschke et al. (2018) also described a similar upregulation of various complement factors in the NaIO_3_ model [25]. They found an accumulation of C1q/C3/C4 at the level of the photoreceptor outer segments. A comparable complement accumulation was observed in the retina of 26-month-old (aged) mice [26]. Luo et al. showed *c1s/c4* expression in the mouse retina, including RPE/choroid under physiologic conditions and a significant increase after treatments with inflammatory cytokines, including interferon (IFN)-γ or tumour necrosis factor (TNF)-α [27].

Activation of the alternative pathway is driven by CFB, which was upregulated on the RNA level, as well as with Simple Western on the protein level after NaIO_3_ treatment. These results were comparable with published data from other groups regarding the detection of CFB under aged and inflammatory conditions [26,27]. The cell-type specific complement expression in healthy and diseased mouse retina was already shown by us in another retinal degeneration mouse model [21].

In the context of retinal complement activation, the complement inhibitor CFH has a pivotal role during pathological events. Single nucleotide polymorphisms in the CFH gene are associated with an increased risk for AMD, and studies with *cfh*^−/−^ mice have shown a pathological accumulation of debris in CFH-deficient RPE cells [26,28]. Here, we found no significant changes in the *cfh* mRNA expression but detected a considerable drop in the protein level. As the CFH protein has been mainly associated with the basal side of RPE cells [29], there is the possibility that the protein originates from the bloodstream. As the levels of the complement components are very high in the serum, and it is known that NaIO_3_ compromises the blood–retina barrier, we cannot exclude that at least some of the detected proteins stem from the blood circulation. However, as shown by us earlier, the Bruch’s membrane is thickened but still intact after the NaIO_3_ treatment [11]. This was also underscored by the fact that we could not detect any mouse IgG in the retina. Furthermore, the fact that the mRNA levels are upregulated in the ocular tissue itself—in some cases, to a very high level (16-fold increase for C3)—implies the presence of a tissue-specific activation cascade.

### 3.3. Inflammatory Processes Induced by NaIO_3_

The NaIO_3_-induced destruction of the retina is mediated by necrosis/necroptosis and, therefore, induces local inflammation [11]. Notably, the opsonisation of dying cells by complement components leads to clearance of the debris by the phagocytes. This can be associated with other inflammatory processes, e.g., immune cell activation and cytokine/chemokine increase, which can further aggravate the retinal degenerative processes. Importantly, the serum cytokine/chemokine levels were not significantly altered in this study. Similar observations were made by others [25,30]. However, we detected increased retinal cytokine/chemokine expression levels in the NaIO_3_ model. Coincidently, aged mice also showed an increased retinal CCL-2 secretion in a previous study [26]. In addition, to upregulated chemokines, the mRNA of *il-1β* was also increased. In accordance with the previous publications, IL-1β is the main downstream effector of the inflammasome and involved in NaIO_3_ pathogenesis [13,30,31]. The inflammasome is also known to activate caspase 1, which has been found significantly upregulated in NaIO_3_-mediated human RPE cell death [32]. The detected chemokines are directly involved in the recruitment of immune cells to the site of degeneration, as we have also shown with a staining for monocytes and microglia.

## 4. Materials and Methods

### 4.1. NaIO_3_ Treatment

Experiments were conducted with 6 to 8-week-old male and female C57BL/6J mice (Charles River Laboratories, Sulzfeld, Germany). The mice were kept in individually ventilated cages (IVC; Tecniplast, Gams, Switzerland) at a temperature of 22 °C, humidity 50% and a 12-h/12-h light cycle with food and water ad libitum. A sterile 1% NaIO_3_ solution (Sigma-Aldrich, Buchs SG, Switzerland) was injected once intravenously (i.v.) via the tail vein. The mice received either 35-mg/kg NaIO_3_ for RNA sequencing or 50-mg/kg NaIO_3_ for all the other experiments. The controls received i.v. the respective volume of 0.9% NaCl (B. Braun Melsungen AG, Melsungen, Germany; 100 μL). The eyes were enucleated three or ten days after treatment, whereas the serum was prepared after ten days only. The experiments were approved by the commission for involving animals in research of the Canton of Bern, Switzerland (BE8/18) and in accordance with the European Community Council Directive 2010/63/EU and the ARVO Statement for the Use of Animals in Ophthalmic and Vision Research. 

### 4.2. Retinal Morphometric Analysis

A morphometric analysis was performed on H&E (a haematoxylin (Mayer’s hemalum, Cat. # 09249.0500; Merck, Darmstadt, Germany) and eosin (Cat. # X883.2; Roth, Arlesheim, Switzerland) stained cross-section of murine eyeballs. To assess the number of photoreceptor cells, the nuclei per longitudinal stretches of 60 µm were counted manually at intervals of 250 µm using ImageJ software (Version 1.51n; National Institutes of Health, Bethesda, MD, USA).

### 4.3. RNA Sequencing

The sensory retina and RPE were harvested separately and immediately frozen in liquid nitrogen. The samples were kept at −80° C until RNA extraction with the RNAeasy Mini QIAcube kit (Cat. # 74116; Qiagen, Hilden, Germany) in the QIAcube (Cat. # 9001293; Qiagen). The samples were homogenised in the RLT buffer from the kit with the addition of 1% β-Mercaptoethanol (Cat. # 63689; Merck, Darmstadt, Germany) inside a TissueLyser II (Cat. # 1102664; Qiagen) with the addition of a stainless-steel bead, 5 mm in diameter (Cat. #69989; Qiagen). RNA quality control was performed on an Experion Automated Electrophoresis station (Cat. # 7007010; Bio-Rad Laboratories, Hercules, CA, USA), and the Experion RNA HighSens Analysis kit (Cat. # 7007105, Bio-Rad Laboratories, Munich, Germany) according to the manufacturer’s protocols. 

The samples were prepared with the HiSeq PE Rapid Cluster Kit v2 and HiSeq Rapid SBS Kit v2 with 50 cycles (Cat. PE-402-4002 and FC-402-4022, Illumina, San Diego, CA, USA) and sequenced with the HiSeq 2500 system (Cat. #SY-401-2501; Illumina) on rapid run mode. The sequencing reads were mapped on the mouse genome draft GRCm38 using the program STAR [33] and the Ensembl gene models (version 92). Genes with a low mean number of counts across the samples (<150) were removed, and about 14k genes passed this filtering step. A differential gene expression analysis was performed in R using the limma library [34,35,36]. The gene counts were converted into log2 CPM (log2 counts per million), and the dataset was assessed for inter- and intragroup variability and expression correlation profiles (pairwise Pearson correlation) in order to evaluate the data quality.

### 4.4. Quantitative Real-Time Polymerase Chain Reaction (qPCR)

After enucleation, the retinas of both mouse eyes were dissected, and the mRNA was isolated using a NucleoSpin RNA Kit (Macherey-Nagel, Düren, Germany). Eluted mRNA was reverse-transcribed to cDNA using the QuantiTect Reverse Transcription Kit (Qiagen, Hilden, Germany). To analyse the gene expression, qPCR was conducted using a Rotor-Gene Q Real-time PCR Cycler, Rotor Gene SYBR Green Kit (all Qiagen) and primers (Table 1; Metabion, Planegg, Germany). For amplification, 40 cycles consisting of an annealing temperature at 60 °C for 10 s was applied. The expression of *isocitrate dehydrogenase 3 subunit beta (idh3b)* was used for normalisation of the gene expression [21]. The fold change in the expression between the control and treatment groups was subsequently calculated employing the ΔΔCt method and transformed to the logarithm to base 2. The data are shown as log2(ΔΔCt).

### 4.5. Simple Western™ Protein Analysis

Frozen retinas were thawed on ice and transferred to Precellys tubes (soft tissue homogenising CK14, Cat. # KT03961-1-203.0.5; Bertin Technologies SAS, Montigny-le-Bretonneux, France) and 1x RIPA buffer (#20-188; Sigma, Burlington, VT, USA). Protease-inhibitor mix (1x cOmplete EDTA-free Mini, Cat. # 11836170001; Roche Diagnostics, Rotkreuz, Switzerland) was added for homogenisation in the Precellys 24 system (Bertin Technologies SAS) for 15 s with 5500 rpm twice, with intermittent cooling on ice. The homogenates were transferred into Protein Lo-Bind tubes (Cat. # 0030108434; Eppendorf, Hamburg, Germany) and centrifuged at 16,000× *g* at 4 °C for 3 min. The supernatant was collected in fresh Protein Lo-Bind tubes and processed for the protein concentration analysis with BCA Protein Assay Kit Pierce™ (Cat. # 23225; Thermo Fisher Scientific, Waltham, MA, USA) and afterwards diluted to 1 mg/mL in aLISA buffer (AlphaLisa Immunoassay Buffer 10x, Cat. # AL000F; PerkinElmer, Waltham, MA, USA). A total volume of 35 µL was prepared, and the samples were aliquoted into 6-µL aliquots into Simport strips (amplitude PCR reaction strips 8× 0.2 mL, attached domed cap #T320-3N Simport, Beloeil, QC, Canada) and frozen at −80 °C until use. As a positive control, normal mouse serum (Cat. # NMS, Complement-Technology, Tyler, TX, USA) was also prepared as a 1:50 dilution in aLISA buffer and frozen at −80 °C. Protein samples and primary and secondary antibodies were loaded into a 96-well plate and analysed by capillary electrophoresis using a 12–230-kDa separation matrix on a Peggy Sue device, as described by the provider (Cat. # 004-800, ProteinSimple, San Jose, CA, USA). The working solutions were diluted with Mastermix (according to the preparation datasheet of the provider) so that the final concentration for the lysates was 0.8 mg/mL.

The following commercially available antibodies were tested for specificity and optimised for the system: rabbit anti-C3 (Cat. #ab200999; Abcam, Cambridge, UK), goat anti-C1s (Cat. #A302; Quidel Corporation, San Diego, CA, USA), rat anti-C4 (Cat. #ab11863, Abcam), goat anti-CFB (Cat. #A311; Quidel Corporation), sheep anti-CFH (Cat. #AF4999; R&D Systems, Minneapolis, MN, USA), rabbit anti-GAPDH (Cat. #G9545; Sigma-Aldrich), anti-goat HRP (Cat. #043-522-2; ProteinSimple, San Jose, CA, USA), anti-sheep HRP (Cat. #HAF016; R&D Systems), anti-rabbit HRP (Cat. #040-0656; ProteinSimple) and anti-rat HRP (Cat. #HAF005; R&D Systems).

### 4.6. Western Blot

Protein extraction from the retinal tissue was carried out using T-PER buffer (Thermo Fisher Scientific) with the addition of 1% protease and 1% phosphatase inhibitor (Sigma-Aldrich). Protein samples were separated by SDS-PAGE using denatured, reducing conditions. The proteins were transferred onto a PVDF membrane. The membrane was blocked with blocking buffer (5% bovine serum albumin (BSA) and 0.1% Tween 20 in Tris-buffered saline) and primary antibodies (goat anti-C3d antibody (Cat. #AF2655; R&D Systems) or rabbit anti-GAPDH antibody (Cat. #CSB-PA00025A0Rb; Cusabio Technology LLC, Houston, TX, USA)) were added overnight. Detection was performed using secondary antibodies (anti-goat IgG-POD #SBA-6442-05, anti-rabbit-POD #711-546-152 (Dianova GmbH, Hamburg, Germany)) and the Lumi-Light Western Blotting Substrate (Cat. # 12015200001; Sigma-Aldrich) and imaged by the FluorChem FC2 Imaging System (AppliChem, Darmstadt, Germany). Western blot images were edited in Adobe Photoshop CS6 (San Jose, CA, USA) and analysed for signal intensity using ImageJ software (National Institutes of Health).

### 4.7. Immunohistochemistry Staining

Determination of the complement expression in the retina and RPE was performed as previously described [37,38]. Paraformaldehyde-fixed, cryoprotected and embedded eyes were cut into 20-µm slices, permeabilised, blocked with 5% goat or donkey serum/0.1% Tween20 in phosphate-buffered saline (PBS) and incubated with primary antibodies specific for C3d (Cat. #AF2655; R&D Systems), C1s (Cat. #14554--1--AP; Proteintech, Rosemont, IL, USA), C4 (Cat. #A205; Complement Technologies, Tyler, TX, USA), IBA (Cat. #019-19741; Wako Chemicals, Neuss, Germany) and glial fibrillary acidic protein (GFAP; G3893, Sigma-Aldrich) diluted in blocking buffer. The sections were labelled with fluorescence-conjugated secondary antibodies (anti-mouse Cy5 (Cat. #115-607-072; Dianova) & anti-goat Cy3 (Cat. #705-165-147; Dianova) and anti-rabbit AF-488 (Cat. #A-21206; Invitrogen)) and DAPI nucleic acid stain (Cat. # 62248c; Invitrogen). All images were taken with a confocal microscope (VisiScope; Visitron Systems, Puchheim, Germany).

### 4.8. C3a ELISA

Nunc-Immuno MaxiSorp 96-well plates were coated with anti-mouse C3a antibody (Cat. #558250; 3 µg/mL, BD Biosciences) in a phosphate buffer (pH 6.5) at 4 °C overnight. The plates were blocked using 1% BSA in PBS. Sera of the control and NaIO_3_-treated mice (1:8000 and 1:16,000), as well as normal mouse serum (1:1000–1:64,000 for the standard curve) were incubated in a sample buffer (0.1 mg/mL nafamostat mesylate in 1% BSA/PBS). Detection was performed with the anti-mouse C3a-biotin antibody (Cat. #558251, 2 µg/mL in PBS, BD Biosciences), streptavidin-HRP and 3,3′,5,5′-tetramethylbenzidine (TMB). The optical density (absorption) was measured photometrically at 450 nm using VarioScan Flash (Thermo Fisher).

### 4.9. Multiplex Cytokine ELISA

The customised ProcartaPlex was performed according to manufacturer’s instructions (Invitrogen, Carlsbad, CA, USA). In brief, the magnetic beads for murine CCL3, CCL4, CCL5, IL-1β and VEGF-A were incubated with 1:2 diluted serum samples overnight. An antigen-specific biotinylated detection antibody mixture was applied following the streptavidin–phycoerythrin solution as a detection reagent. The assay was measured on Luminex™ MAGPIX™ (Austin, TX, USA) to determine the mean fluorescence intensity (MFI) for each chemokine or cytokine.

### 4.10. Statistical Analysis

Data were analysed statistically using GraphPad Prism 6 Software. To determine the statistical differences between the two groups unpaired, the nonparametric Mann–Whitney *t*-test was used. To compare three or more different treatment groups, ordinary one-way analysis of variance (ANOVA) with Tukey’s multiple comparisons was performed. For a statistical analysis of the morphometrical data, a two-way ANOVA with Bonferroni post-tests was performed. *p*-values < 0.05 were considered statistically significant.

## 5. Conclusions

Retinal degeneration induced by NaIO_3_ in mice can serve as a model mimicking retinal inflammatory sequence. The data could help to decipher possible mechanisms of retinal degenerative and inflammatory processes in human patients. We showed that soluble immune proteins, complement proteins and cytokines/chemokines are time-dependently altered locally in this mouse model. However, using a mouse model to understand the pathologies in a different species always has limitations. Therefore, our data, first of all, showed the situation in the murine environment. Nevertheless, as the induced retinal degeneration follows the main cell death pathways such as necrosis and apoptosis, a generalisation for mammalian retina might be appropriate. In sum, this body of evidence supports the hypothesis that a local immune response plays a pivotal role in retinal degeneration.

## Figures and Tables

**Table 1:** qPCR primer.

Gene	Sequence 5′–3′	Reference	T_m_ (°C)
*c1qb*	F: CTCTGGGCTCTGGGAATCCA	Primerblast	61
	R: CCTCAGGGGCTTCCTGTGTA		61
*c1s*	F: CCCTGTAGCCACTTCTGCAA	34	60
	R: GGGCAGTGAACACATCTCCA		60
*c3*	F: AGCCCAACACCAGCTACATC	34	60
	R: GAATGCCCCAAGTTCTTCGC		60
*c3ar1*	F: GTTTGCATGGAAGGCTGCTC	Primerblast	60
	R: AGGTTGCTTTTAGTGGGTGGC		61
*c4*	F: TCTGAAGCCTCCAACGTTCC	34	60
	R: TGGGATGGGGAAGGAAATGC		60
	R: TCCTGGTCAGGAGAGCAAGT		60
*cfb*	F: GGTGCCTCACCAACTTGATT	34	58
	R: CTTGGTGTTGGTCCCTGACT		60
*cfh*	F: AAAAACCAAAGTGCCGAGAC	20	57
	R: GGAGGTGATGTCTCCATTGTC		58
*cfp*	F: AGGTGCAAAGGCCTACTTGG	20	60
	R: TGACCATTGTGGAGACCTGC		60
*idh3b*	F: GCTGCGGCATCTCAATCT	20	58
	R: CCATGTCTCGAGTCCGTACC		60

## Data Availability

Data is contained within the article or supplementary material.

## Supplementary Materials

Supplementary materials are available online at https://www.mdpi.com/article/10.3390/ijms22179218/s1.

## References

[1] Wong W.L., Su X., Li X., Cheung C.M.G., Klein R., Cheng C.-Y., Wong T.Y. (2014). Global Prevalence of Age-Related Macular Degeneration and Disease Burden Projection for 2020 and 2040: A Systematic Review and Meta-Analysis. Lancet Glob. Health.

[2] Chiras D., Kitsos G., Petersen M.B., Skalidakis I., Kroupis C. (2015). Oxidative Stress in Dry Age-Related Macular Degeneration and Exfoliation Syndrome. Crit. Rev. Clin. Lab. Sci..

[3] Lashkari K., Teague G.C., Beattie U., Betts J., Kumar S., McLaughlin M.M., López F.J. (2020). Plasma Biomarkers of the Amyloid Pathway Are Associated with Geographic Atrophy Secondary to Age-Related Macular Degeneration. PLoS ONE.

[4] Pujol-Lereis L.M., Schäfer N., Kuhn L.B., Rohrer B., Pauly D. (2016). Interrelation Between Oxidative Stress and Complement Activation in Models of Age-Related Macular Degeneration. Adv. Exp. Med. Biol..

[5] Datta S., Cano M., Ebrahimi K., Wang L., Handa J.T. (2017). The Impact of Oxidative Stress and Inflammation on RPE Degeneration in Non-Neovascular AMD. Prog. Retin. Eye Res..

[6] Ambati J., Atkinson J.P., Gelfand B.D. (2013). Immunology of Age-Related Macular Degeneration. Nat. Rev. Immunol..

[7] Reisenhofer M.H., Balmer J.M., Enzmann V. (2017). What Can Pharmacological Models of Retinal Degeneration Tell Us?. Curr. Mol. Med..

[8] Kannan R., Hinton D.R. (2014). Sodium Iodate Induced Retinal Degeneration: New Insights from an Old Model. Neural Regen. Res..

[9] Hanus J., Anderson C., Sarraf D., Ma J., Wang S. (2016). Retinal Pigment Epithelial Cell Necroptosis in Response to Sodium Iodate. Cell Death Discov..

[10] Enzmann V., Row B.W., Yamauchi Y., Kheirandish L., Gozal D., Kaplan H.J., McCall M.A. (2006). Behavioral and Anatomical Abnormalities in a Sodium Iodate-Induced Model of Retinal Pigment Epithelium Degeneration. Exp. Eye Res..

[11] Balmer J., Zulliger R., Roberti S., Enzmann V. (2015). Retinal Cell Death Caused by Sodium Iodate Involves Multiple Caspase-Dependent and Caspase-Independent Cell-Death Pathways. Int. J. Mol. Sci..

[12] Li Y., Reca R.G., Atmaca-Sonmez P., Ratajczak M.Z., Ildstad S.T., Kaplan H.J., Enzmann V. (2006). Retinal Pigment Epithelium Damage Enhances Expression of Chemoattractants and Migration of Bone Marrow-Derived Stem Cells. Investig. Ophthalmol. Vis. Sci..

[13] Moriguchi M., Nakamura S., Inoue Y., Nishinaka A., Nakamura M., Shimazawa M., Hara H. (2018). Irreversible Photoreceptors and RPE Cells Damage by Intravenous Sodium Iodate in Mice Is Related to Macrophage Accumulation. Investig. Ophthalmol. Vis. Sci..

[14] Hwang N., Kwon M.-Y., Woo J.M., Chung S.W. (2019). Oxidative Stress-Induced Pentraxin 3 Expression Human Retinal Pigment Epithelial Cells Is Involved in the Pathogenesis of Age-Related Macular Degeneration. Int. J. Mol. Sci..

[15] Hadziahmetovic M., Pajic M., Grieco S., Song Y., Song D., Li Y., Cwanger A., Iacovelli J., Chu S., Ying G.-S. (2012). The Oral Iron Chelator Deferiprone Protects Against Retinal Degeneration Induced through Diverse Mechanisms. Transl. Vis. Sci. Technol..

[16] Bhutto I.A., Ogura S., Baldeosingh R., McLeod D.S., Lutty G.A., Edwards M.M. (2018). An Acute Injury Model for the Phenotypic Characteristics of Geographic Atrophy. Investig. Ophthalmol. Vis. Sci..

[17] Yang Y., Ng T.K., Ye C., Yip Y.W.Y., Law K., Chan S.-O., Pang C.P. (2014). Assessing Sodium Iodate-Induced Outer Retinal Changes in Rats Using Confocal Scanning Laser Ophthalmoscopy and Optical Coherence Tomography. Investig. Ophthalmol. Vis. Sci..

[18] Franco L.M., Zulliger R., Wolf-Schnurrbusch U.E.K., Katagiri Y., Kaplan H.J., Wolf S., Enzmann V. (2009). Decreased Visual Function after Patchy Loss of Retinal Pigment Epithelium Induced by Low-Dose Sodium Iodate. Investig. Ophthalmol. Vis. Sci..

[19] Machalińska A., Lejkowska R., Duchnik M., Kawa M., Rogińska D., Wiszniewska B., Machaliński B. (2014). Dose-Dependent Retinal Changes Following Sodium Iodate Administration: Application of Spectral-Domain Optical Coherence Tomography for Monitoring of Retinal Injury and Endogenous Regeneration. Curr. Eye Res..

[20] Anderson D.H., Radeke M.J., Gallo N.B., Chapin E.A., Johnson P.T., Curletti C.R., Hancox L.S., Hu J., Ebright J.N., Malek G. (2010). The Pivotal Role of the Complement System in Aging and Age-Related Macular Degeneration: Hypothesis Re-Visited. Prog. Retin. Eye Res..

[21] Pauly D., Agarwal D., Dana N., Schäfer N., Biber J., Wunderlich K.A., Jabri Y., Straub T., Zhang N.R., Gautam A.K. (2019). Cell-Type-Specific Complement Expression in the Healthy and Diseased Retina. Cell Rep..

[22] Mulfaul K., Ozaki E., Fernando N., Brennan K., Chirco K.R., Connolly E., Greene C., Maminishkis A., Salomon R.G., Linetsky M. (2020). Toll-like Receptor 2 Facilitates Oxidative Damage-Induced Retinal Degeneration. Cell Rep..

[23] Chen M., Muckersie E., Robertson M., Forrester J.V., Xu H. (2008). Up-Regulation of Complement Factor B in Retinal Pigment Epithelial Cells Is Accompanied by Complement Activation in the Aged Retina. Exp. Eye Res..

[24] Grossman T.R., Carrer M., Shen L., Johnson R.B., Hettrick L.A., Henry S.P., Monia B.P., McCaleb M.L. (2017). Reduction in ocular complement factor B protein in mice and monkeys by systemic administration of factor B antisense oligonucleotide. Mol. Vis..

[25] Katschke K.J., Xi H., Cox C., Truong T., Malato Y., Lee W.P., McKenzie B., Arceo R., Tao J., Rangell L. (2018). Classical and Alternative Complement Activation on Photoreceptor Outer Segments Drives Monocyte-Dependent Retinal Atrophy. Sci. Rep..

[26] Chen H., Liu B., Lukas T.J., Neufeld A.H. (2008). The Aged Retinal Pigment Epithelium/choroid: A Potential Substratum for the Pathogenesis of Age-Related Macular Degeneration. PLoS ONE.

[27] Luo C., Chen M., Xu H. (2011). Complement Gene Expression and Regulation in Mouse Retina and Retinal Pigment Epithelium/choroid. Mol. Vis..

[28] Coffey P.J., Gias C., McDermott C.J., Lundh P., Pickering M.C., Sethi C., Bird A., Fitzke F.W., Maass A., Chen L.L. (2007). Complement Factor H Deficiency in Aged Mice Causes Retinal Abnormalities and Visual Dysfunction. Proc. Natl. Acad. Sci. USA.

[29] Fett A.L., Hermann M.M., Muether P.S., Kirchhof B., Fauser S. (2012). Immunohistochemical Localization of Complement Regulatory Proteins in the Human Retina. Histol. Histopathol..

[30] Ma W., Zhang Y., Gao C., Fariss R.N., Tam J., Wong W.T. (2017). Monocyte Infiltration and Proliferation Re-establish Myeloid Cell Homeostasis in the Mouse Retina Following Retinal Pigment Epithelial Cell Injury. Sci. Rep..

[31] Sachdeva M.M., Cano M., Handa J.T. (2014). Nrf2 Signaling Is Impaired in the Aging RPE given an Oxidative Insult. Exp. Eye Res..

[32] Mao X., Pan T., Shen H., Xi H., Yuan S., Liu Q. (2018). The Rescue Effect of Mesenchymal Stem Cell on Sodium Iodate-Induced Retinal Pigment Epithelial Cell Death through Deactivation of NF-κB-Mediated NLRP3 Inflammasome. Biomed. Pharmacother..

[33] Dobin A., Davis C.A., Schlesinger F., Drenkow J., Zaleski C., Jha S., Batut P., Chaisson M., Gingeras T.R. (2013). STAR: Ultrafast Universal RNA-Seq Aligner. Bioinformatics.

[34] Ritchie M.E., Phipson B., Wu D., Hu Y., Law C.W., Shi W., Smyth G.K. (2015). Limma powers differential expression analyses for RNA-sequencing and microarray studies. Nucleic Acids Res..

[35] Law C.W., Chen Y., Shi W., Smyth G.K. (2014). Voom: Precision weights unlock linear model analysis tools for RNA-seq read counts. Genome Biol..

[36] Phipson B., Lee S., Majewski I.J., Alexander W.S., Smyth G.K. (2016). Robust hyperparameter estimation protects against hypervariable gens and improves power to detect differential expression. Ann. Appl. Stat..

[37] Jabri Y., Biber J., Diaz-Lezama N., Grosche A., Pauly D. (2020). Cell-Type-Specific Complement Profiling in the ABCA4 Mouse Model of Stargardt Disease. Int. J. Mol. Sci..

[38] Schäfer N., Grosche A., Schmitt S.I., Braunger B.M., Pauly D. (2017). Complement Components Showed a Time-Dependent Local Expression Pattern in Constant and Acute White Light-Induced Photoreceptor Damage. Front. Mol. Neurosci..

