# Supplementary material for: Sodium Iodate-Induced Degeneration Results in Local Complement Changes and Inflammatory Processes in Murine Retina
Source: Int J Mol Sci. 2021 Aug 26;22(17):9218. doi: 10.3390/ijms22179218 (PMC8431125; doi:10.3390/ijms22179218)
Supplement: Supplementary file 1 [file ijms-22-09218-s001.zip › ijms-1354987-supplementary.pdf]

Supplementary Materials

Sodium Iodate-Induced Degeneration Results in Local Complement Changes and Inflammatory Processes in Murine Retina

Anne Enzbrenner [1], Rahel Zulliger [2], Josef Biber [3], Ana Maria Quintela Pousa [4,5], Nicole Schäfer [1], Corinne Stucki [2], Nicolas Giroud [2], Marco Berrera [2], Elod Kortvely [2], Roland Schmucki [2], Laura Badi [2], Antje Grosche [3], Diana Pauly [1,6,*,†] and Volker Enzmann [4,5,†]

1 Department of Ophthalmology, University Hospital Regensburg, 93053 Regensburg, Germany; a.enzbrenner@googlemail.com (A.E.); Nicole.Schaefer@klinik.uni-regensburg.de (N.S.)
2 Roche Pharma Research & Early Development, Roche Innovation Center Basel, F. Hoffmann-La Roche Ltd., 4070 Basel, Switzerland; rahel.zulliger@roche.com (R.Z.); corinne.stucki@roche.com (C.S.); nicolas.giroud@roche.com (N.G.); marco.berrera@roche.com (M.B.); elod.koertvely@roche.com (E.K.); roland.schmucki@roche.com (R.S.); laura.badi@roche.com (L.B.)
3 Department of Physiological Genomics, Biomedical Center, Ludwig-Maximilians-University Munich, 82152 Planegg-Martinsried, Germany; Josef.Biber@bmc.med.lmu.de (J.B.); Antje.Grosche@bmc.med.lmu.de (A.G.)
4 Department of Ophthalmology, University Hospital of Bern, 3010 Bern, Switzerland; quintelapousa@gmail.com (A.M.Q.P.); volker.enzmann@insel.ch (V.E.)
5 Department of Biomedical Research, University of Bern, 3010 Bern, Switzerland
6 Experimental Ophthalmology, University Marburg, 35043 Marburg, Germany
* Correspondence: diana.pauly@uni-marburg.de
† Contributed equally.

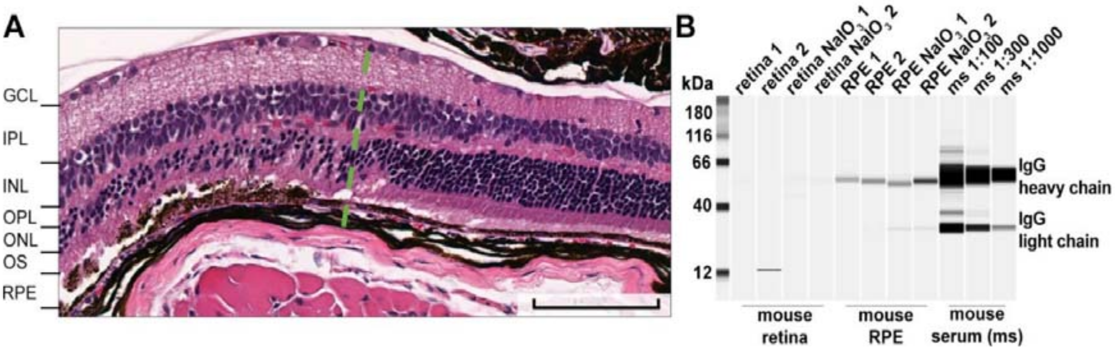

Figure S1. (**A**) Patchy RPE loss and photoreceptor degeneration in the $NaIO_3$-induced damage area (left). The dashed line depicts the transition to the area with physiological morphology (right). H&E staining of murine retinal cross-section three days after $NaIO_3$ treatment. Scale bar: 100 µm. (**B**) No mouse IgG were detected in the retinal tissue using Simple WesternTM technology and anti-mouse IgG-HRP (mouse serum as positive control) three days after $NaIO_3$ treatment.

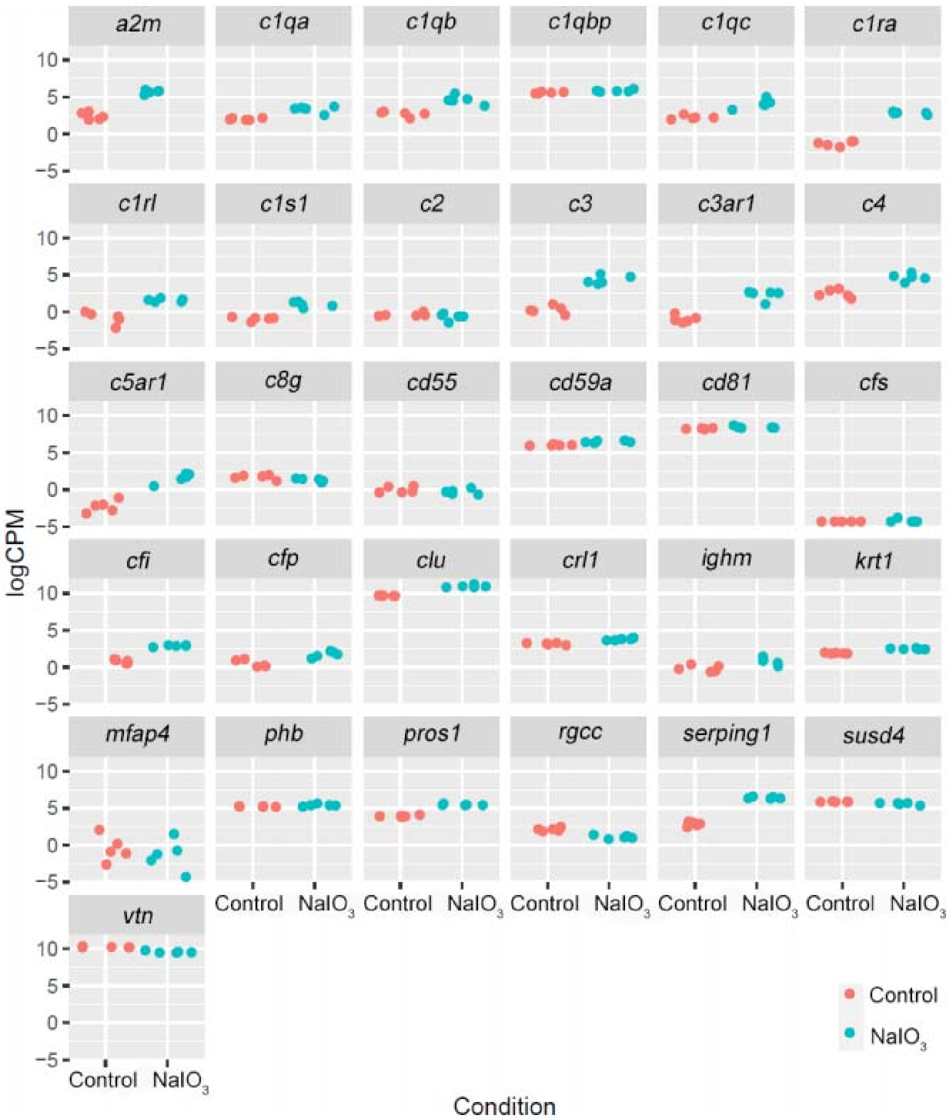

Figure S2. Transcript quantity of components of the complement pathway (GO: 0006956) investigated in control (blue) or $NaIO_3$-treated (red) retina using RNA-Seq analysis three days after $NaIO_3$ treatment.

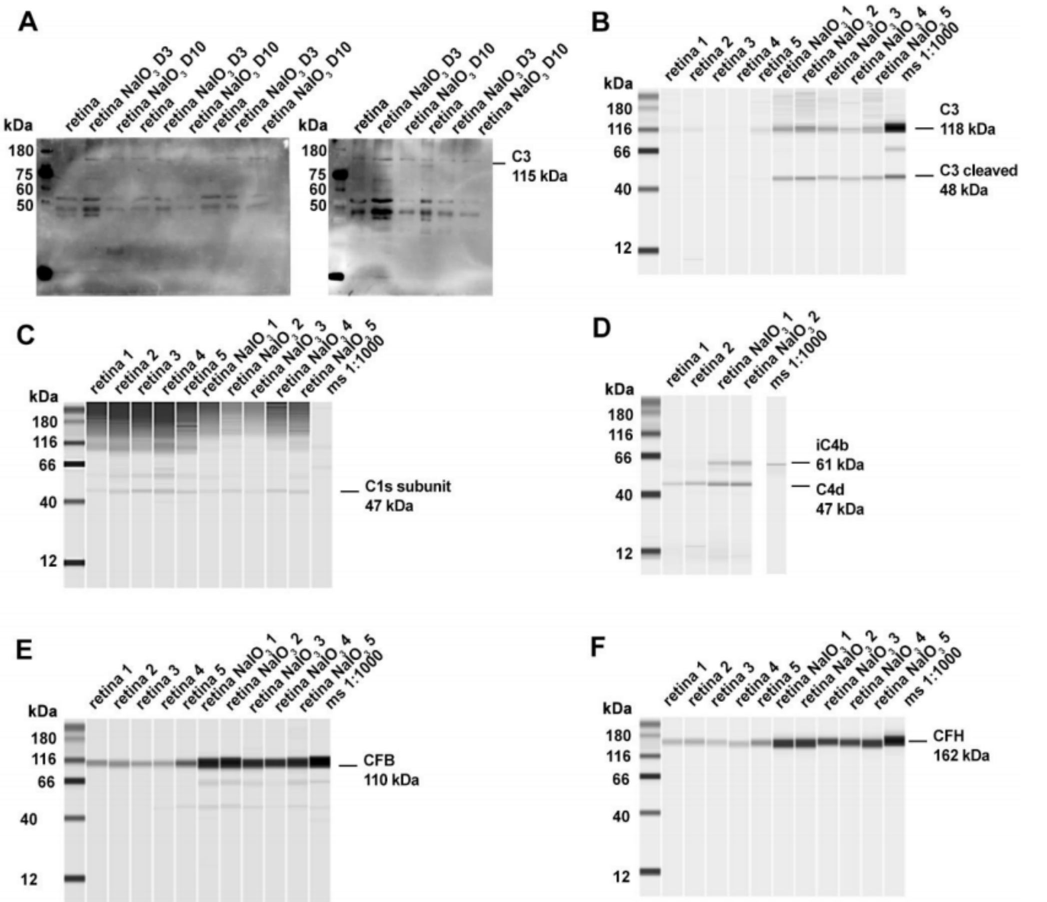

Figure S3. (**A**) Full Western blot is shown for C3 detection in untreated retinas and three as well as ten days after $NaIO_3$-treatment. (**B**–**F**) Full Simple Western™ blots are shown for C3, C1s, C4, CFB and CFH detection in untreated retinas and three days after $NaIO_3$-treatment.

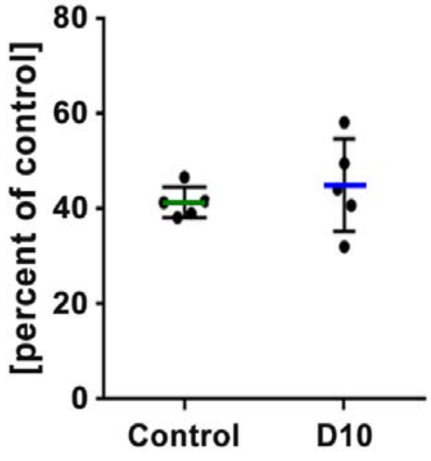

Figure S4. $NaIO_3$-treatment does not alter systemic complement activation. Complement activation product, anaphylatoxin C3a, is analyzed in an ELISA comparing serum samples of untreated and $NaIO_3$-treated mice, ten days post-treatment. Systemic C3a levels of treated mice do not differ significantly from those of untreated animals.
